# Left and right brain-oriented hemisity subjects show opposite behavioral preferences

**DOI:** 10.3389/fphys.2012.00407

**Published:** 2012-11-02

**Authors:** Bruce E. Morton

**Affiliations:** Department of Biochemistry and Biophysics, John A. Burns School of Medicine (JABSOM), University of HawaiiHonolulu, HI, USA

**Keywords:** brain behavioral laterality, executive asymmetry, hemisity and personality, hemisty and gender

## Abstract

**Introduction:** Recently, three independent, intercorrelated biophysical measures have provided the first quantitative measures of a binary form of behavioral laterality called “Hemisity,” a term referring to inherent opposite right or left brain-oriented differences in thinking and behavioral styles. Crucially, the right or left brain-orientation of individuals assessed by these methods was later found to be essentially congruent with the thicker side of their ventral gyrus of the anterior cingulate cortex (vgACC) as revealed by a 3 min MRI procedure. Laterality of this putative executive structural element has thus become the primary standard defining individual hemisity. **Methods:** Here, the behavior of 150 subjects, whose hemisity had been calibrated by MRI, was assessed using five MRI-calibrated preference questionnaires, two of which were new. **Results:** Right and left brain-oriented subjects selected opposite answers (*p* > 0.05) for 47 of the 107 “either-or,” forced choice type preference questionnaire items. The resulting 30 hemisity subtype preference differences were present in several areas. These were: (1) in logical orientation, (2) in type of consciousness, (3) in fear level and sensitivity, (4) in social-professional orientation, and (5) in pair bonding-spousal dominance style. **Conclusions:** The right and left brain-oriented hemisity subtype subjects, sorted on the anatomical basis of upon which brain side their vgACC was thickest, showed 30 significant differences in their “either-or” type of behavioral preferences.

## Introduction

Hemisphericity (Bogen, 1969), is a once popular but now discredited (Beaumont et al., 1984) concept of behavioral laterality where each individual's thinking and behavioral style was supposed to occupy a unique point upon a gradient between right and left brain extremes. This definition was fundamentally flawed because the behavioral outcome of most subjects was near the middle of the gradient, giving these studies essentially no analytical or predictive power. However, over the subsequent quarter of a century since the demise of hemisphericity, evidence has been accumulating that robust different right or left brain-oriented thinking and behavioral styles do indeed exist. What was required to reveal these differences was the replacement of the gradient context of “hemisphericity” by the binary context of “hemisity” where each individual is either left or right brain-oriented with no intermediates possible. This was accomplished in part by the use of forced choice binary questions.

Based on this binary concept, three independent, but highly intercorrelated biophysical methods (dichotic deafness, two-hand line bisection, two-hand mirror tracing) have shown persistent significant differences between individuals (Morton, 2001, 2002, 2003a,b,c). The results of these methods were confirmed and amplified by the MRI demonstration that the ventral region of the dorsal anterior cingulate cortex was thickest on the same side of the subject's brain as their biophysically predetermined hemisity [in 130 of 133 subjects (98%), Morton and Rafto, 2010].

With the ability to accurately determine the right or left brain individual hemisity subtype identity in hand, the time has come to answer some very interesting questions: Do these biophysically identified right and left hemisity subtype individuals differ significantly in their behavioral preferences? And if so, specifically how? To approach this question, the same 133 multiply determined hemisity subtype subjects were assessed, based upon their answers to five behavioral preference questionnaires, three published (Zenhausern, 1978; Morton, 2002, 2003c), and two new ones reported here. A surprisingly large number of these “either-or” choices were answered oppositely by the two hemisity subtype subjects. Morton (2003d) has reported that a hemisity sorting occurred as college freshmen migrated through college, graduate school, and into 15 professions.

## Methods

### Subjects

Subjects (*n* = 133) were volunteers from the University of Hawaii community, 44.0 years mean age, ± 14.5 years S.D. Of these, 47.3% (63) were female, 11% (15) claimed left-handedness, and 77% (103) were Caucasian, the rest being predominantly Asian. Based upon an earlier MRI study (Morton and Rafto, 2010), there were 45.1% (60) right brain-oriented persons (RPs) composed of 48.3% (29) right brain-oriented females (RFs) and 51.7% (31) right brain-oriented males (RMs). The 54.9% (73) left brain-oriented persons (LPs) consisted of 47.9% (35) left brain-oriented females (LFs) and 52.1% (38) left brain-oriented males (LMs). The Committee of Human Studies of the University of Hawaii Institutional Review Board had earlier approved all appropriate elements of this unfunded research.

### The five behavioral preference questionnaires

Subjects completed the five preference questionnaires. In Table 1, these were compared for reliability and for overall correlation with the predetermined subject hemisity subtype. They were Zenhausern's (1978) “Preference Questionnaire,” Morton's “Polarity Questionnaire (PQ)” (Morton, 2002) and the Asymmetry Questionnaire (AQ) (Morton, 2003c). The new Binary Questionnaire (BQ) (Appendix 1) and new Hemisity Questionnaire (HQ) (Appendix 2) were also filled out.

**Table 1:** **Overall correlations and reliability of preference questionnaire scores with predetermined subject hemisity subtype**.

**Preference questionnaires**	***r* (Pearsons)**	***p***	***N***	% yield^*^	**alpha Cron-bach's**
**CORRELATIONS OF PRE-ASSIGNED HEMISITY SUBTYPES WITH:**					
Zenhausern's Preference Questionnaire	0.24	0.008	119	35	0.37
Polarity Questionnaire	0.57	0.000	132	82	0.57
Asymmetry Questionnaire	0.48	0.000	111	60	0.64
Binary Questionnaire	0.43	0.000	112	30	0.66
Hemisity Questionnaire	0.53	0.000	79	48	0.65
Best Hand Test (R−L)	0.37	0.000	143		
Mirror Tracing Test (R/L)	0.50	0.000	116		
Dichotic Deafness Test (R−L/R+L)	0.34	0.000	109		

* % yield refers to the percentage of questionnaire statements that were significantly associated with subject neuroanatomical hemisity. Pre-assigned hemisity subtype, direction of asymmetry of the ventral gyrus of the anterior cingulate cortex.

### Statistics

The internal reliabilities of the questionnaires were assessed by Cronbach's alpha. Pearson correlations were used to assess the association between overall scores on the five questionnaire measures (as well as continuous measures derived from each of the three biophysical hemisity measures) and with the pre-assigned hemisity subtype of the 133 subjects. One-Way analysis of variance were used to determine which items in the five questionnaires differed significantly between right and left hemisity subtype participants using SPSS statistical software. Of the 107 questionnaire items, 32 non-overlapping items showed significant differences in preference between left and right brain-oriented subjects (Table 4). Four items were collapsed into two binary concepts. The 30 binary items for which these MRI-calibrated hemisity subtype subjects differed were sorted into five arbitrary hemisity behavioral categories in Table 5.

## Results

Overall correlations of the five preference questionnaires with predetermined subject hemisity subtype (Table 1) ranged from −0.24 for Zenhausern's Preference Questionnaire, to −0.57 for the PQ, with the AQs, BQs, and HQs falling in between at Pearson's *r* values of = −0.48, −0.43, and −0.53, respectively. Of these five questionnaires, the 11 true false statement PQ was the most efficient solo instrument with 82% of the items (9/11) showing significant associations with predetermined hemisity. Cronbach's alpha values of the tests (Table 1) ranged from 0.37 to 0.66, indicating that more than one of these questionnaires would be required to determine individual hemisity, if this were to be the sole method of assessment. As a comparison, the three biophysical hemisity assays were also included at the bottom of the table.

The associations among the six secondary hemisity instruments (three biophysical and three psychometric), as well as hemisity, determined by the average hemisity outcome of the combination of these six binary assays (Hemisity-6), by the two additional questionnaires (BQ and HQ), and hemisity determined by anterior cingulate MRI asymmetry were examined using cross tabulations and chi square analyses (Table 2). As may be seen, the pre-assigned hemisities were very strongly associated with hemisity classifications based on the three biophysical methods, all five questionnaires, and the combination of the six secondary methods (Hemisity-6). All three of the biophysical methods were strongly associated with each of the others. The summary measure of hemisity (Hemisity-6) was strongly associated with all of the other hemisity measures. The PQs, AQs, and HQs were strongly associated with each of the other classifications. The BQ classification was significantly associated with nine of the other hemisity classifications, while the associations with two others [Mirror Tracing Test, Preference Test (PT)] reached only trend levels of significance. The classification based on Zenhausern's earlier PT (1978) was significantly associated with six of the other hemisity classifications. But, as expected, it was associated with the BQ and HQ classifications at only a trend level of significance, and was not associated with classifications based on the Best Hand Test, Mirror Tracing Test, or AQ.

**Table 2:** **Cross-tabulations involving anterior cingulate asymmetry and hemisity categories**.

		**ACC thick**		**BHT**		**MTT**		**Dichotic**		**Polarity Q**		**Pref Test**		**Asym Q**		**Total**
		**L**	**R**	**L**	**R**	**L**	**R**	**L**	**R**	**L**	**R**	**L**	**R**	**L**	**R**	**N**
Best Hand Test^*^	L	78^**^	22													
	R	28	72													
	χ^2^ (*p*)	35.54 (0.000)														
Mirror Tracing Test^*^	L	77	23	67	33											
	R	27	73	29	71											
	χ^2^ (*p*)	27.86 (0.000)		23.73 (0.000)												
Dichotic Deafness	L	78	22	68	32	71	29									
	R	25	75	37	63	39	61									
	χ^2^ (*p*)	29.75 (0.000)		14.07 (0.000)		13.90 (0.000)										
Polarity Q	L	89	11	70	30	81	19	74	26							
	R	30	70	37	63	36	64	43	57							
	χ^2^ (*p*)	44.97 (0.000)		19.67 (0.000)		33.49 (0.000)		13.27 (0.000)								
Preference Test	L	71	29	59	41	62	38	68	32	52	48					
	R	41	59	45	55	48	52	42	58	35	65					
	χ^2^ (*p*)	11.27 (0.001)		2.70 (0.100)		2.59 (0.107)		7.90 (0.005)		4.18 (0.04)						
Asymmetry Q	L	81	19	72	28	69	31	75	25	65	35	60	40			
	R	33	67	36	64	41	59	41	59	22	78	47	53			
	χ^2^ (*p*)	26.06 (0.000)		17.82 (0.000)		10.21 (0.001)		13.76 (0.000)		25.84 (0.000)		2.30 (0.13)				
Hemisity-6	L	89	11	80	20	85	15	87	13	77	23	68	32	76	24	74
	E	77	23	69	31	40	60	45	55	20	80	64	36	45	55	12
	R	7	93	15	85	20	80	19	81	7	93	27	73	10	90	60
	χ^2^ (*p*)	92.82 (0.000)		81.84 (0.000)		67.34 (0.000)		64.03 (0.000)		89.05 (0.000)		23.16 (0.000)		57.17 (0.000)		
Binary Q	L	77	23	79	21	63	37	68	32	62	38	62	38	83	17	52
	R	37	63	33	67	47	53	49	51	27	73	46	54	14	86	61
	χ^2^ (*p*)	18.14 (0.000)		28.28 (0.000)		3.25 (0.07)		4.09 (0.04)		17.53 (0.000)		3.58 (0.058)		65.22 (0.000)		
Hemisity Q	L	87	13	74	26	93	7	71	29	65	35	56	44	76	24	38
	R	29	71	43	57	47	53	41	59	26	74	34	66	18	82	41
	χ^2^ (*p*)	26.67 (0.000)		7.76 (0.005)		15.72 (0.000)		5.73 (0.02)		11.26 (0.001)		3.26 (0.07)		21.48 (0.000)		
Total N		79	67	76	68	68	48	76	68	54	79	61	59	53	59	146

* Best Hand Test and Mirror Tracing Test are phase corrected, and the laterality designation (L or R) refers to hemisity, not hand skill.

** Percentage of subjects with either L or R column asymmetry, within the hemisity groups defined by row variables.

A continuous measure of neuroanatomical asymmetry derived from predetermined hemisity was significantly correlated with continuous measures of asymmetry derived from the Best Hand Test, Mirror Tracing Test, Dichotic Deafness Test, PQs, AQs, BQs, and HQs, and the PT (Table 3). All of the continuous measures derived from these hemisity determination methods (degree of leftness) were significantly intercorrelated, with the exception of the correlations between the Mirror Tracing Test and BQ, and the PT and HQ. Thus, for the most part, all nine of these asymmetries were strongly associated with each of the others, as only two of the 36 correlations among these nine variables failed to reach significance. The marking of some of the data with a negative sign is an artifact of the original definitions of the instruments involved.

**Table 3:** **Correlations among continuous hemisity measures**.

	**BHT**	**MTT**	**DDT**	**PQ**	**PT**	**AQ**	**BQ**	**HQ**
MTT	0.41							
DDT	−0.29	−0.39						
PQ	0.33	0.41	−0.36					
PT	0.20^*^	0.20^*^	−0.23^*^	0.29				
AQ	0.33	0.30	−0.37	0.59	0.35			
BQ	0.23^**^	*0.15*	−0.26^**^	0.58	0.33	0.81		
HQ	0.35^**^	0.42	−0.31^*^	0.50	*0.18*	0.63	0.59	
ACC	−0.40	−0.50	0.32	−0.53	−0.27^**^	−0.45	−0.42	−0.47

* p < 0.05,

** p < 0.01, italics p > 0.05, all others, regular typeface p < 0.001.

BHT, Best Hand test; MTT, Mirror Tracing Test; DDT, Dichotic Deafness Test; PQ, Polarity Questionnaire; PT, Zenhausern's Preference Test; AQ, Asymmetry Questionnaire; BQ, Binary Questionaire; HQ, Hemisity Questionnaire; ACC, anterior cingulate thickness laterality quotient (R−L)/(R+L).

Table 4 lists the 47 questionnaire items which showed a significant difference between participants with Left > Right vs. those with Right > Left ventral gyrus of the anterior cingulate cortex (vgACC) thickness. Since the BQ and the HQ contained items that were also included (or very similar in content) in Zenhausern's PT, PQ and AQs, 15 redundant items were deleted, and two items were collapsed into a single binary item, leaving 30 binary items showing significant associations with anterior cingulate asymmetry. For example, asterisks in the lower part of Table 4 indicate which of the items were unique to the new BQs and HQs. Attempts to derive item groupings empirically using factor analysis yielded 17 factors for the 47 significant items and 12 factors for the 30 non-redundant items, which were difficult to interpret. Therefore, these items were grouped into five domains, on a priori grounds (Table 5). These were: (1) logical orientation, (2) type of consciousness, (3) fear level and sensitivity, (4) social-professional orientation, (5) pair bonding-spousal dominance style.

**Table 4:** **Forty-seven items showing significant differences between left vs. right greater vgACC thickness**.

**Number**	**Item**	***F***	**df**	**mean sq.**	***p***
PQ1	When I become upset, after cooling down I don't need to talk, I need to be alone.	45.00	1,132	8.448	0.000
PQ3	I would rather maintain and use good old solutions than find new better ones.	4.54	1,132	0.737	0.035
PQ5	I am comfortable and productive in the presence of disorder and disorganization.	10.75	1,132	2.513	0.001
PQ6	I find it very difficult to tolerate when my mate (or “important other”) becomes defiant to me in private. >	16.80	1,132	3.593	0.000
PQ7	I don't need a lot of physical contact from my mate.	9.66	1,132	2.060	0.002
PQ8	I like daily small reassurances of my mate's love more than monthly large rewards. >	7.68	1,132	1.625	0.006
PQ9	I tend not to be very romantic or sentimental.	6.76	1,132	1.528	0.010
PQ10	I am more strict than lenient with our children (or I would be if I had children). >	4.10	1,132	1.002	0.045
PQ11	Given the opportunity, I am more of an early morning person than a late night person.	6.58	1,132	1.573	0.011
PT1	How often are your decisions based upon objective facts rather than feelings? (L)	5.61	1,119	14.104	0.020
PT2	Are you psychic (i.e., can you read minds, can you predict well)? >	5.94	1,119	139.44	0.016
PT9	How vivid are your daydreams? >	9.61	1,119	55.712	0.002
PT11	Do you remember your dreams often? >	4.17	1,119	168.54	0.013
PT14	Do you use a playful approach to solving problems? >	11.77	1,119	43.452	0.001
PT18	How often foes your thinking consist of words? (L)	5.48	1,119	68.939	0.021
PT19	How often does your thinking consist of mental pictures or images? >	6.09	1,119	42.994	0.015
AQ1	I often talk about my and other's feelings of emotion. > Vs. I tend to avoid talking about emotional feelings.	13.47	1,112	3.045	0.000
AQ5	I think and listen interactively-vocally, and talk a lot. > Vs. I think and listen quietly, keep my talk to a minimum.	5.23	1,112	1.253	0.024
AQ6	I don't read other people's mind very well. vs. I am very good at knowing what others are thinking. >	8.25	1,112	1.934	0.005
AQ7	I see the big picture (project beyond data, can predict). > vs. I am analytical (stay within the limits of the data).	12.07	1,112	1.953	0.001
AQ8	I tend to be independent, hidden, private, and indirect. vs. I tend to be interdependent, open, public, and direct. >	17.67	1,112	3.882	0.000
AQ10	I need to be alone and quiet when upset. vs. I need closeness and to talk things out when upset. >	19.79	1,112	3.804	0.000
AQ11	I praise others, and also work for praise from others. > vs. I do not praise others, nor need the praise of others	4.14	1,112	0.540	0.044
AQ13	I seek frank feedback from others. > vs. I avoid seeking evaluation by others	5.02	1,112	0.948	0.027
AQ14	I often feel my mate talks too much. vs. I feel my mate doesn't talk or listen to me enough. >	9.05	1,112	2.065	0.003
HQ1	After upset, I need aloneness and quiet, vs. I need closeness/to talk. >	19.33	1,79	3.844	0.000
HQ5	My mate talks too much, vs. my mate doesn't talk or listen enough. >	18.38	1,79	3.822	0.000
HQ6	I talk about feelings, > vs. I avoid talking about my or other's emotions.	21.09	1,79	4.188	0.000
HQ7	I'm independent, private, indirect, vs. interdependent, open and direct. >	22.85	1,79	4.488	0.000
HQ9	My daydreams are not vivid, vs. My daydreams are vivid. >	7.68	1,79	1.766	0.007
HQ11^*^	I am more conservative and cautious, vs. innovative and bold. >	6.25	1,79	1.478	0.015
HQ15^*^	When relating to others am myself sensitive, vs. intense. >	5.91	1,79	1.346	0.017
HQ17^*^	To self-medicate, I would choose depressants vs. stimulants. >	4.89	1,79	1.131	0.031
HQ20^*^	I tend to take the blame, > vs. I try to avoid taking the blame.	11.71	1,79	2.439	0.001
HQ21	My emotions are overwhelming, vs. my emotions are endurable. >	7.11	1,79	1.630	0.009
BQ4	I see the big picture, can predict, > vs. I am analytical within limits of that data.	12.46	1,113	1.998	0.001
BQ6^*^	I imagine, convert concepts to contexts > vs. I abstract objects into concepts.	4.22	1,113	1.021	0.042
BQ9^*^	I concentrate on many things at once, vs. I concentrate on one thing at a time. >	4.71	1,113	1.105	0.032
BQ15	I don't read other people's mind well, vs. I know what others are thinking. >	8.77	1,113	2.044	0.004
BQ20	I talk about my and other's feelings, > vs. I avoid talking about emotions.	14.16	1,113	3.183	0.000
BQ21	I am hidden, independent, indirect, vs. I am open, interdependent, direct. >	18.53	1,113	4.050	0.000
BQ22	I seek frank feedback from others, > vs. I avoid seeking evaluation by others.	5.29	1,113	0.991	0.023
BQ25	When I am upset, I need to be alone quiet vs. I need closeness and to talk. >	20.99	1,113	4.037	0.000
BQ26^*^	I often take blame myself or apologize, > vs. I usually avoid taking the blame.	9.68	1,113	1.822	0.002
BQ30	I praise others and also work for praise, > vs. I do not praise, nor need it.	4.48	1,113	0.583	0.037
BQ34^*^	I'm observant of my surroundings, > vs. I think and ignore my surroundings.	5.63	1,113	1.207	0.019
BQ35	I feel my mate talks too much, vs. my mate doesn't talk or listen enough. >	9.56	1,113	2.168	0.003

**Table 5:** **Thirty binary behavioral correlates of hemisity**.

**Left brain-oriented persons**	**Right brain-oriented persons**
**LOGICAL ORIENTATION**	
Analytical (stays within the limits of the data)	Sees the big picture (projects beyond data, predicts)
Uses logic to convert objects to literal concepts	Imagines, converts concepts to contexts or metaphors
Decisions based on objective facts	Decisions based on feelings, intuition
Uses a serious approach to solving problems	Use a playful approach to solving problems
Prefers to maintain and use good old solutions	Would rather find better new solutions
**TYPE OF CONSCIOUSNESS**	
Daydreams are not vivid	Has vivid daydreams
Doesn't often remember dreams	Remembers dreams often
Thinking often consists of words	Thinking often consists of mental pictures or images
Can easily concentrate on many things at once	Tends to concentrate on one thing in depth at a time
Comfortable and productive with chaos	Slowed by disorder and disorganization
Often thinking tends to ignore surroundings	Observant and in touch with surroundings
Often an early morning person	Often a late night person
**FEAR LEVEL AND SENSITIVITY**	
Conservative, cautious	Innovative, bold
Sensitive in relating to others	Intense in relating to others
Tend to avoid talking about emotional feelings	Often talks about own and others feelings of emotion
Suppresses emotions as overwhelming	Seeks to experience and express emotions more deeply
Would self-medicate with depressants	Would self-medicate with stimulants
**SOCIAL AND PROFESSIONAL ORIENTATION**	
Does not read other people's mind very well	Good at knowing what others are thinking
Thinks-listens quietly, keeps talk to minimum	Thinks-listens interactively, talks a lot
Independent, hidden, private, and indirect	Interdependent, open, public, and direct
Does not praise others nor work for praise	Praises others and works for praise
Avoids seeking evaluation by others	Seeks frank feedback from others
Usually tries to avoid taking the blame	Tends to take the blame, blames self, or apologizes
**PAIR-BONDING AND SPOUSAL DOMINANCE STYLE**	
Tolerates mate defiance in private	Finds it difficult to tolerate mate defiance in private
After an upset with spouse, needs to be alone	After upset with spouse, needs closeness and to talk
Needs little physical contact with mate	Needs a lot of physical contact with mate
Tends not to be very romantic or sentimental	Tends to be very romantic and sentimental
Prefers monthly large reassurances of love	Likes daily small assurances of mate's love
Often feels mate talks too much	Feels my mate doesn't talk or listen enough
Lenient parent, kids tend to defy	Strict, kids obey and work for approval

## Discussion

Recently, it became possible to segregate people into an anatomical-behavioral dichotomy, called hemisity. This was made possible by use of three biophysical methods (Morton, 2001, 2002, 2003a,b). As the result of further extension of this research, hemisity can now be determined, based upon which side of an individual's vgACC is thickest, as revealed by a 3 min MRI scan (Morton and Rafto, 2010). These four methods were earlier used to separate a subset of the subjects of this study into two groups: LPs and RPs. This enabled us to approach a most interesting question about hemisity: what thinking and behavioral style differences exist between right and left brain-oriented hemisity subtypes.

The goal of this research was to inquire whether RPs in general had any behavioral preferences in common that were different or opposite to those of LPs. To this end, five behavioral preference type questionnaires, three containing non-overlapping “either-or” forced choice type of binary statements (Morton, 2002, 2003c) and two new ones reported here (Appendix 1 and 2), were administered to the MRI-calibrated members of these two hemisity subgroups. The outcome was that the right and left brain-oriented hemisity groups choose statistically significant opposite responses for 47 of the 107 questionnaire items. Eliminating redundant items and collapsing two items into one binary concept yielded 30 non-redundant behavioral characteristics. These behavioral preference differences occurred in five areas: (1) logical orientation, (2) type of consciousness, (3) fear level and sensitivity, (4) social-professional orientation, and (5) pair bonding-spousal dominance style (Table 5). There follows a brief discussion of how these results may be related to many previously studied psychological, personality, health and mental health topics.

### Logical orientation

#### Local—global

Consistent with the much discussed local vs. global anatomy and putative opposite function of the left and right cerebral hemispheres (review by Ivry and Robertson, 1998), the LPs in this study showed a local processing bias, compared to the global bias of the RPs. If this dichotomy associated with hemisity is indeed mediated by hemispheric asymmetry of local vs. global processing, it is hypothesized that future studies should find a local processing advantage in subjects with left hemisity, and a global processing advantage in subjects with right hemisity.

#### Logical—intuitive

In keeping with earlier studies (Prifitera, 1981), since the hemisity subtypes show analytic/logical vs. gestalt/holistic preferences, it is also hypothesized that right hemisity will be associated with higher scores on intuition, feeling, perceiving, and/or extraversion, while left hemisity will be associated with higher scores on sensing, thinking, judging, and/or introversion on Jungian personality types, as measured by the Myers-Briggs Type Indicator (MBTI). In this semantic context, “feeling” is a gut estimate subjective opinion, while “sensing” is a quantitative analysis based upon measurement. This may be tested by the greater ability of RPs to accurately assess values objectively from only qualitative data. Without measurement, LPs tend to be less accurate in such estimation.

#### Verbalizer—visualizer

Here, left hemisity is associated with a preference for thinking in words, while right hemisity is associated with a preference for thinking in pictures, suggesting that hemisity is related to the “verbalizer—visualizer” distinction. Consistent with many earlier studies (Richardson, 1977, 1978; Montgomery and Jones, 1984), it is hypothesized that right hemisity will be associated with a predominantly visualizer style, while left hemisity will be associated with a predominantly verbalizer style.

### Type of consciousness

#### Transliminality

Similarly, LPs and RPs were significantly different in their choices regarding the elements considered to be associated with consciousness. That LPs indicated having non-vivid daydreams and rare recall of dreams, places them opposite to RPs with more vivid daydreams and common recall of dreams. This suggests that LPs have a greater barrier to the unconscious and that the RPs in contrast are the more transliminal. Transliminality is the relative tendency for psychological material to cross thresholds into or out of consciousness. Transliminality is strongly associated with sleep-related experiences. Scores on the Iowa Sleep Experiences Scale were significantly correlated with several measures of schizotypy, measures of dream recall frequency, openness to experience, fantasy proneness, and absorption (Fassler et al., 2006). It is hypothesized that right hemisity will be associated with higher scores on the Iowa Sleep Experiences Scale. It is further hypothesized that right hemisity will be associated with higher scores on the Revised Transliminality Scale (Thalbourne et al., 1997).

#### Absorption

It is interesting that LPs seem to be able to concentrate on many things at once and thrive on chaos, while RPs appear to think deeply on only one thing at a time in a form of absorption (Tellegen and Atkinson, 1974) and appear to be distracted by disorder. It is hypothesized that right hemisity will be associated with higher scores on measures of absorption and hypnotizability.

#### Mindfulness

The association of right hemisity with a greater awareness of one's surroundings suggests a possible association between right hemisity and the trait of mindfulness. An operational definition of mindfulness as “the awareness that emerges through paying attention on purpose, in the present moment and non-judgmentally to the unfolding of experience moment by moment” was proposed by Kabat-Zinn (2003). Attention and cognition are critically involved in mindfulness (Malinowski, 2008), which entails self-regulation of attention and orientation toward one's experiences. It is hypothesized that right hemisity will be associated with higher scores on measures of trait mindfulness.

#### Fantasy proneness

Fantasy proneness is also associated with vividness of visual imagery (van de Ven and Merckelbach, 2003). It is hypothesized that right hemisity will be associated with higher scores on measures of fantasy proneness.

#### Morningness—eveningness

The association of morningness with left hemisity and eveningness with right hemisity here is consistent with other literature correlates of chronotype too numerous to cite here, such as (Adan and Almirall, 1992 or Muro et al., 2009). Eveningness is significantly associated with extraversion, impulsivity, externalizing behaviors, lower self-control, higher novelty seeking, greater sensation seeking, greater risk-taking propensity, greater openness to experience, a greater sense of humor, and more frequent nightmares. Eveningness is also associated with greater depressive symptoms, subclinical manic symptoms, and a greater tendency to bipolar disorder. Both depressive and manic tendencies are components of transliminality.

Morningness is associated with greater introversion, conscientiousness, and anxiety than eveningness. Morningness is associated with higher baseline arousal levels than eveningness, as indicated by higher mean waking and daytime cortisol levels, higher levels of energetic arousal, and lower immunity. It is predicted that right hemisity will be associated with eveningness, while left hemisity will be associated with morningness, as measured by the Morningness–Eveningness Questionnaire (Horne and Ostberg, 1976).

#### Alexithymia

Left hemisity is associated with a tendency to avoid talking about feelings. A syndrome named “alexithymia” has been described as characterized by an inability to verbally describe feelings, flattened affect, inability to recognize emotions in others, absence of fantasies and dreaming, preoccupation with minute details of external events, somatic complaints, and withdrawn personality traits (Sifneos et al., 1977). Studies of commisurotomized patients suggest that the dissociation between affect and cognition seen in alexithymia is related to a functional deconnection of the hemispheres (Hoppe and Bogen, 1977). A clinical study of a patient with agenesis of the corpus callosum revealed severe alexithymia (Buchanan et al., 1980). Increased size of the corpus callosum may be associated with greater interhemispheric communication (Christman, 1995). Right hemisity is associated with a larger area of the corpus callosum (Morton and Rafto, 2006), and with lower levels of alexithymia. It is predicted that left hemisity will be associated with higher scores on the Toronto Alexithymia Scale (Bagby et al., 1994).

#### Repressors

A repressive defense style, i.e., preferential use of the defense mechanism of repression has been found to be associated with relatively greater left hemisphere activation (Waldinger and Van Strien, 1995). A repressive coping style has been psychometrically identified in terms of high scores on the Marlowe-Crowne Social Desirability Scale (MCSDS) and low scores on anxiety scales (Tomarken and Davidson, 1994). It was associated with inhibition of the perception of threat, repression of experiences of negative affect, and use of a self-regulatory style that promotes self-esteem. This involved a self-serving attributional style, a self-serving hindsight bias, impaired memory of negative self-relevant feedback and negatively toned autobiographical events. It also showed a preference for defense mechanisms characterized by the inhibition of interpersonal conflict and ambivalent or negative emotions, and by selective accentuation of the positive (Schwartz, 1990). Tomarken and Davidson (1994) and several others found that compared to low anxious and high anxious subjects, repressors had significantly greater left frontal EEG activation, heightened autonomic responsivity and systolic blood pressure. The repressive coping style may be associated with a functional hemispheric deconnection (Schwartz, 1990). Consistent with the deconnection hypothesis, Davidson (1984) found that repressors showed relative deficits in interhemispheric transfer of negative affective information from the right to the left hemisphere. Thus, both relatively greater left frontal activation and reduced cross-callosal transfer of negative affective information appear to be associated with repressive coping style. It is here predicted that left hemisity will be associated with a repressive style, as indicated by high scores on measures of social desirability, and low scores on measures of anxiety on the MCSDS (Tomarken and Davidson, 1994).

### Fear level and sensitivity

#### Anxiety—confidence

As an element that seemed to underlie many of the choices in this and other sections of the analysis, it appears that the LPs were more anxiety prone than RPs. Here, this was suggested by choices between feeling conservative and cautious vs. innovative and bold. It was also reflected in feeling sensitive vs. bold in personal relations, suggestive of personal dominance issues. Again, LPs preferred to avoid experiencing and talking about their and others emotions while the RPs appeared to revel in doing so. It is hypothesized that left hemisity will be associated with higher scores on anxiety ranking scales such as Spielberger's State-Trait Anxiety Inventory (trait version) and to be more prone to anxiety-based stress-induced opportunistic illnesses, chronic fatigue syndrome, and post-traumatic stress disorder.

### Social and professional orientation

#### Extroversion-introversion

Several of the items in the social and professional orientation cluster suggest an association between left hemisity with introversion and between right hemisity with extraversion. Introversion is associated with greater cerebral blood flow while extraversion with less CBF (Mathew et al., 1984), consistent with higher cortical arousal levels in introverts. Introverts exhibited a non-verbal decoding deficit, relative to extraverts, possibly suggesting relatively lesser right than left hemisphere functioning in introverts (Lieberman and Rosenthal, 2001). Introverts tend to show hyper-anxious Type A behavior that is also associated with a tendency to repress stimulation in order to down regulate excessive baseline arousal (Ludvigh and Happ, 1974), leading them to self-medicate with depressants and consequent proneness to alcoholism (Morton, 2011, 2012). Extroverts, in contrast, to compensate for low baseline arousal, seek out stimulation in order to up regulate cortical arousal to optimum levels. As found here, RPs appear to prefer stimulating substances over depressants (Morton, 2011, 2012). Previously cited studies indicated that higher baseline arousal is also associated with morningness, alexithymia, and a repressive style, all of which are characteristic of left hemisity. Extraversion has been found to be significantly associated with various characteristics associated with transliminality and right hemisity, including creativity and mania/hypomania (Cassano et al., 2009). Extraversion is also associated with higher levels of emotional awareness (Igarashi et al., 2011) and right hemisity is associated with greater emotionality.

#### Theory of mind abilities

Theory of mind is the ability to make inferences about the intentions, feelings, and beliefs (mental states) of others and to use these inferences to predict and control behavior (Premack and Woodruff, 1978). Theory of mind abilities have been found to be associated with various aspects of non-literal language, including metaphor, humor, irony, and sarcasm (Langdon et al., 2002; Channon et al., 2005). Right hemisity is associated with an evidence-based belief in a greater ability to know what others are thinking. Patients with right hemisphere damage have been shown to be impaired in the comprehension of similes, metaphors, proverbs, sarcasm, humor, and other non-literal inferences (Brownell et al., 1990). Both affective prosody and sarcasm perception are thought to depend on the right hemisphere (Shamay-Tsoory et al., 2005). The right frontal lobe, particularly the ventral medial region, is pre-eminently involved in the mediation of theory of mind abilities and self-awareness (Stuss et al., 2001). Impairments on theory of mind tasks have also been found in patients following right hemisphere stroke (Happé et al., 1999). It is hypothesized that right hemisity will be associated with better performance on Theory of Mind tasks.

### Pair bonding and spousal dominance style

#### Attachment

The items included in the pair bonding and spousal dominance cluster refer to behaviors related to attachment style, suggesting that right hemisity is associated with greater activation of the behavioral attachment system, while left hemisity is associated with lower activation of the behavioral attachment system. Theoretical arguments and empirical studies have suggested that attachment behaviors are mediated predominantly by the right hemisphere, particularly the right orbitofrontal cortex (Mohr et al., 2008). As demonstrated by the present data, it is predicted that left hemisity will be associated with an avoidant spousal attachment style, while right hemisity will be associated with a more dominant spousal attachment style.

### Limitations and opportunities

Although Zenhausern's Preference Questionnaire was one of the best from the hemisphericity era instruments, it was substantially weaker than the four other HQs derived from the more recent biophysical methods used here. It employed a Likert (1932) type of rating scale. Because of a tendency to avoid making critical choices by marking intermediate values, this approach may have contributed to the statistical downfall of earlier hemisphericity studies (Beaumont et al., 1984). Generation and use of the other four forced choice binary response HQs used here has markedly improved their reliability. Yet, while raising Cronbach's alpha measures of internal and external reliability from about 0.37 into the 0.54–0.66 range, the latter still leave something to be desired in terms of the 0.9 alpha value commonly accepted as indicative of high specificity. However, the reconceptualization of hemisity as a typological dichotomy suggests that high internal consistency may not be an appropriate measure of psychometric adequacy, as internal consistency is more relevant to measures of trait dimensions. The HQs may be better thought of as taxonic indicators, which frequently include multiple relatively independent items related to taxon membership (e.g., Golden and Meehl, 1979).

The unpublished BQs and HQs (Appendix 1 and 2) were collections of guesses made as to how left brain-oriented individuals might differ. They were included here because they ended up providing several additional items where hemisity subtypes chose opposite answers to a significant extent. They were not as specific as the published PQs and AQs (Morton, 2002, 2003c). Nevertheless, the numbers of subjects in this study enabled the individual items in each of the five questionnaires to provide significant correlations for 47 of the test items (yielding 30 non-redundant binary concepts), thus opening a window to investigating personality differences between right and left brain-oriented individuals. It is expected that more contrasting behaviors of hemisity will be found. It must be stated that the probability that a specific hemisity subtype will have any one of these 30 specific traits is approximately eighty percent. That is, about one in five RPs will be morning larks, while the other four will be night owls.

## Conclusions

Recently, three independent, intercorrelated biophysical measures have provided the first quantitative measures of a binary form of hemisphericity, called “Hemisity,” a term referring to inherent opposite right or left brain-oriented differences in thinking and behavioral styles. The right or left brain-orientation of individuals assessed by these methods was later found to be essentially congruent with the thicker side of their vgACC (Morton and Rafto, 2010). Here, the behavioral preferences of 133 subjects, whose hemisity subtype had been determined repeatedly in previous reports, were assessed using five preference questionnaires, two of which were new. Right and left brain-oriented subjects selected opposite answers (*p* > 0.05) for 47 of the 107 “either-or” type preference questionnaire items, which were reduced to 30 binary preferences by elimination of redundant items. The thirty hemisity subtype preference differences occurred in five general areas: logical orientation, type of consciousness, fear level and sensitivity, social-professional orientation, and pair bonding-spousal dominance style. The right and left brain-oriented hemisity subtype subjects, showed numerous significant differences in their “either-or” type of behavioral preferences, some of which may have physical and mental health consequences.

It is as yet unclear to what extent hemisity traits contribute to the overall personality. This may be difficult to analyze. For example, although many RPs find it difficult to keep quiet, there was a small group of LPs who were very verbose. Similarly, it appears that some physically fearless RPs have social anxieties while many socially adept LPs have anxieties about physical danger. Further research is needed to explore the relationship between hemisity and overall personality, as conceptualized in the Big Five personality theory. Given the observed associations, it is hypothesized that right hemisity will be positively correlated with extraversion and openness to experience, whereas left hemisity is hypothesized to be positively correlated with conscientiousness and negatively correlated with openness to experience.

Perhaps even more difficult will be uncovering the underlying brain basis for these differences. The demonstration of the existence of hemisity opens the doors of explanation beyond sex-gender based personality ideas for many presently confusing characteristic human attitudes and human relations outcomes.

### Conflict of interest statement

The author declares that the research was conducted in the absence of any commercial or financial relationships that could be construed as a potential conflict of interest.

## Appendix 1

### A binary preference questionnaire

Bruce E. Morton, Ph.D., University of Hawaii School of Medicine

Your Name or Number: ______________, Sex, M or F___, Handedness, R or L__, Parental Ethnicity____________.

For each pair of statements, mark an X by the viewpoint that is most like your own.

**Table:**

**Statement A**	**Statement B**
**MEMORY PROCESSING**	
1. I look for differences, separate, and analyze things.	I look for similarities, commonalities, and unify things.
2. I organize parts into the whole (synthetic, creative).	I break the whole into parts (reductive, reductionistic).
3. I manipulate concepts deductively, see important details.	I manipulate contexts inductively and can generalize.
4. I see the big picture (project data beyond, can predict).	I am analytical (stay within the limits of the data).
5. I symbolize and label things: (a symbol = 1000 words).	I visualize and concretize things: (a picture = 1000 words).
6. I imagine, convert concepts into contexts or metaphors.	I use logic, convert objects into literal concepts.
**TYPE OF CONSCIOUSNESS**	
7. I thrive in the early morning.	I am alert in the late evening.
8. I am good at completing things.	I am a strong starter of projects.
9. I can easily concentrate on many things at once.	I tend to concentrate on one thing in depth at a time.
10. I am orderly, organized, and deliberate.	I am disorganized, disorderly, but fast.
11. I am quick-acting in emergency.	I methodically solve problems (process of elimination).
12. I am uncomfortable with chaos, and am slowed by it.	I am comfortable with chaos, am accelerated by it.
13. I have good ideas, not all of which are practical.	I'm very intuitive, insightful about idea applications.
14. I'm self-conscious, feel guilty, and am a poor performer.	I'm not self-conscious, have low guilt, and perform well.
15. I dont read other peoples mind very well.	I am very good at knowing what others are thinking.
16. I feel communication is my source of power and support.	I feel communication is of lesser importance to me.
**FEAR, AROUSAL, SENSITIVITY**	
17. I value tradition, respect authority, and resist change.	I am innovative, question authority, and seek change.
18. I am more radical, daring, and experimental.	I am more conservative, cautious, and avoiding.
19. I tend not to invade other's boundaries.	I may invade other's boundaries.
20. I often talk about my and other feelings of emotion.	I tend to avoid talking about emotional feelings.
21. I tend to be independent, hidden, private, and indirect.	I can be interdependent, open, public, and direct.
22. I seek frank feedback from others.	I avoid seeking evaluation by others.
23. I am comfortable in groups, even adversarial ones.	I am uncomfortable in groups, unless loyal friends.
24. I have an out-of-control temper, but it only lasts minutes.	I can control my anger but I may last for hours.
25. I need to be alone and quiet when I am upset.	I need closeness, to talk things out when Im upset.
26. I often take responsibility, blame myself, or apologize.	I usually avoid taking the blame.
27. I'd rather rationalize a way to be right than be wrong.	I'd rather be wrong than rationalize a way to be right.
**GENERAL BEHAVIORAL STYLE**	
28. I think and listen interactively-vocally, and talk a lot.	I think and listen quietly, keep my talk to a minimum.
29. I tend to use humor to tease or mock the other person.	I often tend to make humor at my own expense.
30. I praise others, and also work for praise from others.	I do not praise others, nor need the praise of others.
31. I'm immediate, thick-skinned, no time for self-analysis.	I'm contemplative, thin skinned, intense self-analysis.
32. I usually design my own outfits of clothing.	I dress for success and wear high status clothing.
33. I'm more interested in objects and things.	I tend to be more interested in people and feelings.
34. I'm very observant and in touch with my surroundings.	I'm often thinking and tend to ignore my surroundings.
35. I often feel my mate talks too much.	I feel my mate doesnt talk or listen to me enough.
36. I am the nurturance-requiring member of a couple.	I am the more nurturing member of a couple.
37. I am a supportive, highly competitive partner.	I am an innovative, directive, yet cooperative partner.
38. I'm strict, my kids obey me, and work for my approval.	I'm not a strict parent and my kids don't obey me well.
39. I don't need a lot of physical closeness from my mate.	I need lots of physical closeness from my mate.
40. I find it intolerable if my mate defies me in private.	I can tolerate it if my mate defies me in private.

%Left Score = Odd As__ + Even Bs__ = ___ x 2.5.

## Appendix 2

### Hemisity questionnaire

Bruce E. Morton, Ph.D., University of Hawaii School of Medicine

Name or I.D. Number________________. Sex___, Age___, Handedness___, Ethnicity of your Mother's family____________, Ethnicity of your Father's family______________.

**Table:**

Write an **A** or **B** for the statement most like you, or most like the way you think.	
___1.	After I have been upset with my mate, **A**. I need to be alone and quiet, vs. **B**. I need closeness and to talk things out. (If you are not currently in such a relationship, imagine how you would feel if you were.)
___2.	If my mate defies me in private, I find it to be, **A**. very difficult to tolerate, **B**. something I can put up with.
___3.	**A**. I am analytical (stay within the limits of the data), vs. **B**. I see the big picture (project beyond the data, can predict).
___4.	Regarding disorder, **A**. I am stressed and slowed by it, vs. **B**. I am comfortable or even accelerated by it.
___5.	**A**. I often feel my mate talks too much, vs. **B**. I feel my mate doesn't talk or listen to me enough.
___6.	**A**. I often talk about my and other's feelings of emotion, vs. **B**. I tend to avoid talking about my or other's emotional feelings.
___7.	**A**. I tend to be independent, hidden, private, indirect, vs. **B**. I tend to be interdependent, open, public, and direct.
___8.	In this country I wish there were, **A**. more high-quality law and order, or **B**. more personal freedom.
___9.	**A**. My daydreams are not vivid, vs. **B**. My daydreams are vivid.
___10.	**A**. My thinking consists of images, vs. **B**. My thinking often consists of words.
___11.	I feel that I am more, **A**. conservative and cautious, vs. **B**. innovative and bold.
___12.	As a parent in a nuclear family, I am, **A**. the most dominant spouse, vs. **B**. the most supportive spouse (If not presently in a spousal relationship, imagine that you were in one.)
___13.	Can you comfortably carry on a conversation with someone in the same room and with another person on the telephone at the same time? **A**. No, vs. **B**. Yes.
___14.	To others my desk might appear to be, **A**. neat, vs. **B**. messy.
___15.	When relating to others I would describe myself as, **A**. sensitive, vs. **B**. intense.
___16.	In terms of my health, **A**. I am almost never ill, vs. **B**. I catch colds, the flu, etc., rather easily.
___17.	If I were to self-medicate with drugs, I would choose, **A**. a depressant such as alcohol or cannabis vs. **B**. a stimulant such as cocaine or amphetamine.
___18.	**A**. I often enjoy chatting with others, vs. **B**. I tend to find social chatter to be somewhat annoying.
___19.	**A**. I don't read other people's minds very well, vs. **B**. I am very good at knowing what others are thinking.
___20.	**A**. I tend to take the blame, vs. **B**. I try to avoid taking the blame.
___21.	**A**. I avoid deeply experiencing or expressing my emotions because they seem so overwhelming that I am afraid I might lose control. **B**. I am not afraid to deeply experience and express my emotions because they are not that overwhelming.

Odd As + Even Bs = Left score/21.
